# A Proof‐of‐Principle Study for *δ* ^15^N Measurements of Aqueous Dissolved Nitrate With a Modified LC‐IRMS Interface

**DOI:** 10.1002/rcm.9950

**Published:** 2024-11-26

**Authors:** Tobias Hesse, Felix Niemann, Shaista Khaliq, Daniel Köster, Julian Enss, Christian K. Feld, Milen Nachev, Klaus Kerpen, Maik A. Jochmann, Torsten C. Schmidt

**Affiliations:** ^1^ Instrumental Analytical Chemistry University of Duisburg‐Essen Essen Germany; ^2^ Probenahmedienst Feststoffe, Ressourcen‐ und Qualitätsmanagement Landesamt für Natur, Umwelt und Verbraucherschutz NRW Duisburg Germany; ^3^ Institut für Arbeitsschutz der DGUV (IFA) Deutsche Gesetzliche Unfallversicherung e.V. (DGUV) Sankt Augustin Germany; ^4^ Aquatic Ecology, Faculty of Biology University of Duisburg‐Essen Essen Germany; ^5^ Centre for Water and Environmental Research University of Duisburg‐Essen Essen Germany

**Keywords:** CSIA, LC‐IRMS, nitrate, S ^15^N‐NO_3_, stable nitrogen isotopes, *δ* ^15^N

## Abstract

**Rationale:**

The analysis of nitrogen isotopes in aqueous dissolved nitrate is an effective method for identifying pollution sources and offers the potential to study the nitrogen cycle. However, the measurement of nitrogen isotope ratios of nitrate still requires extensive sample preparation or derivatization.

**Methods:**

In this study, a modified commercially available liquid chromatography–isotope ratio mass spectrometer (LC‐IRMS) interface is presented that enables automated measurement of *δ* ^15^N signatures from nitrate by online reduction of nitrate in two consecutive steps. First, vanadium(III) chloride is used as a reducing agent to convert NO_3_^−^ to N_*x*_O_y_ under acidic conditions. The mix of nitrogen oxides is then transferred into a stream of helium and reduced to nitrogen (N_2_) analysis gas via a hot copper reactor. Prior to the online conversion of aqueous nitrate into elemental nitrogen, the sample was chromatographically separated from potential matrix effects on a PGC column.

**Results:**

Precision was achieved at a level below 1.4‰ by injecting 10 μL of 50 mg L^−1^ N, using five different nitrate standards and reference materials. These materials spanned a range of more than 180‰ in *δ* ^15^N. To demonstrate the applicability of the method, we measured water samples from an enrichment experiment, where isotopically enriched ammonium chloride was administered into a small river over the course of 2 weeks. In contrary to our expectation, the δ^15^N values of river nitrate showed values between +0.4 ± 0.4‰ and +4.1 ± 0.3‰, varying over a small range of 3.7‰.

**Conclusions:**

Our study showed that the measurement of nitrate nitrogen isotope ratios with a modified LC‐IRMS system is possible but that further modifications and improvements would be necessary for a robust and user‐friendly instrument.

## 1 Introduction

The main inorganic nitrogen species in rivers are ammonium, nitrite, and nitrate [1]. Contamination of water by anthropogenic nitrate has become a global environmental concern, and the measurement of nitrogen isotope ratios of nitrate is an effective method for identifying and differentiating between natural and anthropogenic nitrate sources, and it provides opportunities to study the nitrogen cycle. However, established methods for determining the nitrogen isotopic composition of nitrate at natural abundance levels are demanding and often hinder rapid and reliable measurements required in water monitoring programs. A variety of methods have been developed for the determination of nitrate and nitrite isotope ratios. The Supporting Information provides a brief overview of the methods used.

The initial objective of our study was to develop a compound‐specific liquid chromatography–isotope ratio mass spectrometry (LC‐IRMS) method for the measurement of nitrogen isotope ratios of organic substances.

The method comprises four principal stages: (i) separation of the organic nitrogenous compounds by liquid chromatography; (ii) wet chemical oxidation to NO_3_^−^ using peroxydisulfate under acidic or basic conditions; (iii) NO_3_^−^ reduction by vanadium(III) chloride to gaseous N_x_O_y_, which is transferred into a helium stream through a gas permeable membrane; and (iv) further reduction to N₂ on hot copper wires prior to introduction into an IRMS. To develop such an LC‐IRMS method for the measurement of nitrogen isotope ratios of organic compounds, one problem that had to be solved first was whether nitrogen species formed during the oxidation of organic compounds with peroxydisulfate could be converted into a measurable gas form such as N_2_O, NO, or N_2_ [2, 3]. Therefore, our goal was to develop a method for measuring the nitrogen isotope ratio of nitrate by online conversion to gaseous nitrogen oxide species (N_x_O_y_) and subsequent reduction to elemental nitrogen while maintaining the ability to inject samples in small volumes. Two‐step reduction to N_2_ was chosen instead of one‐step reduction to N_2_O by Ti(III). The reason for this was the original intention to develop an LC‐IRMS method for nitrogen isotope ratio measurements of organic compounds. This development requires a wet chemical oxidation by peroxydisulfate. We retained the conversion to N_2_ to investigate the method with nitrate samples as an important intermediate step for the realization of a general N‐isotope interface for LC‐IRMS. Furthermore, the goal was to achieve a chromatographic separation of nitrate from the injection peak and potentially nitrite. Here, we present the initial findings of standard sample measurements conducted with a modified LC‐IRMS interface and discuss the method limitations and possible options for further improvement and future applications. Additionally, we had the opportunity to test our system with samples from an enrichment experiment conducted in a small sandy river.

## 2 Materials and Methods

### 2.1 Instrumental Setup

**FIGURE 1:**
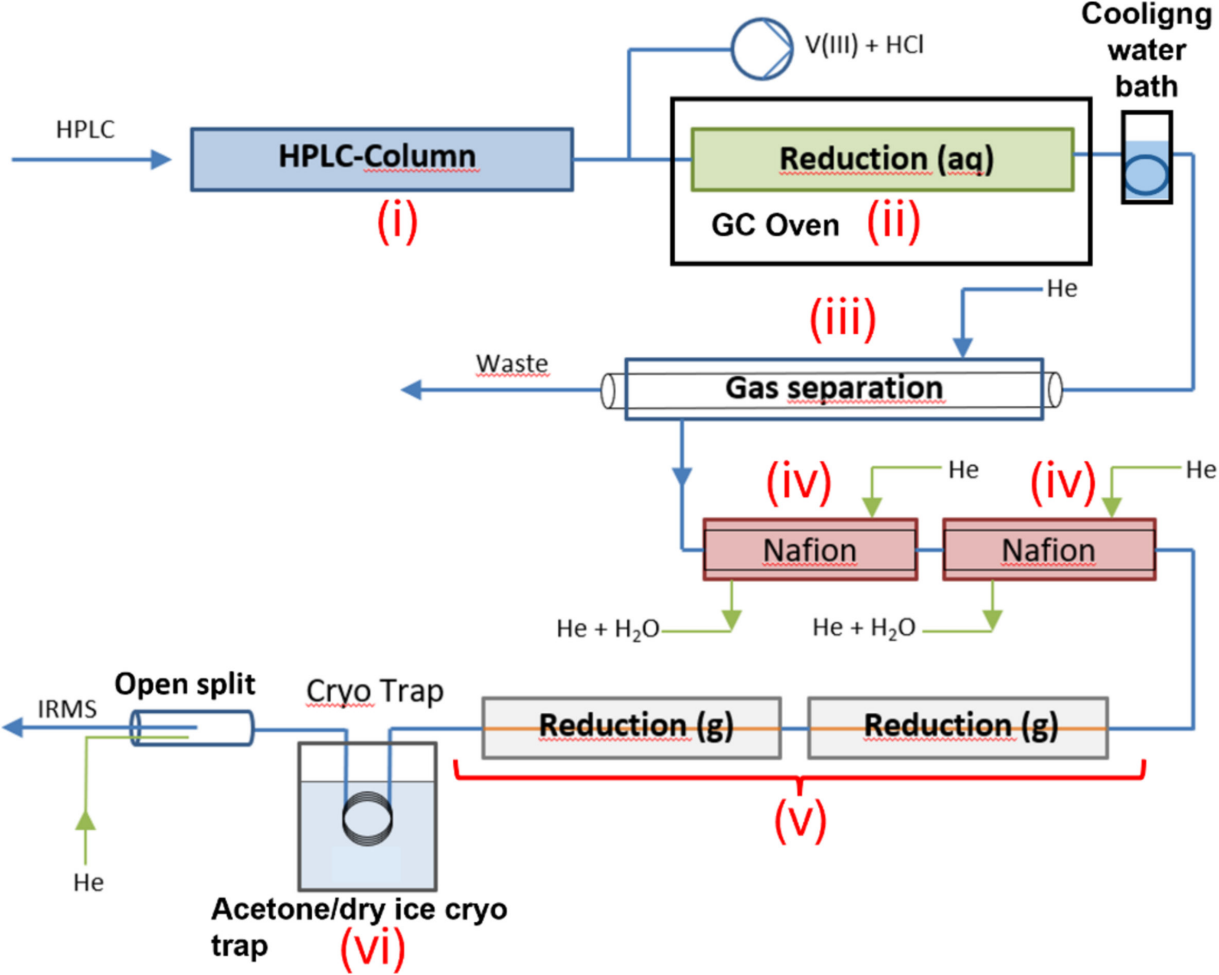
Instrumental setup of the modified LC‐IRMS for the determination of *δ* ^15^N isotope values of nitrate and nitrite. (i) Chromatographic separation of nitrate by ion chromatography, (ii) reduction to N_x_O_y_, (iii) gas separation unit, (iv) gas drying by two Nafion dryer units, (v) reduction reactor, and (vi) acetone/dry ice cryo trap to trap CO_2_. The nitrogen gas was introduced via an open split into the IRMS.

The instrument that we used in our initial proof‐of‐principle study was a modified commercially available LC IsoLink interface (Thermo Fisher Scientific Inc., Bremen, Germany) (see Figure 1). In a preliminary step (i), nitrate was chromatographically separated from the injection peak and potential matrix interferences on a porous graphitic carbon (PGC)‐HPLC column. In a second step, nitrate was reduced by a V(III)Cl_3_ solution to N_x_O_y_ with nitric oxide (NO) being the main product formed. Nitrogen oxides were separated from the eluent by membrane pervaporation (iii). After Nafion drying (iv), N_x_O_y_ and NO were reduced to N_2_ gas by a copper‐filled ceramic reduction tube in a furnace (v) [4, 5]. Following reduction to N_2_, carbon dioxide was removed by a cryo trap with a slurry of acetone/dry ice (vi) before the N_2_ entered the IRMS via an open split.

### 2.2 Chemicals and Solutions

Vanadium(III) chloride (97%, Merck, Darmstadt, Germany) was used to prepare solutions of 0.015 M V(III)Cl_3_ in 0.3 M HCl (36.5%–38%, Alfa Aesar, Kandel, Germany). In order to preserve the HPLC pumps, which were constructed from stainless steel and were susceptible to high chloride concentrations, we did not utilize excessive amounts of reducing agents. Heated hydrochloric acid is highly corrosive and can irreparably damage the stainless‐steel reactor in the LC‐IRMS interface; therefore, a deactivated silica capillary was used (see below). The reduction of nitrate to nitric oxide with vanadium(III) chloride follows the chemical reaction [6]:

(1) $${\mathrm{NO}}_{3}^{-}+3V^{3+}+4H^{+}\to \mathrm{NO}+3V^{4+}+2H_{2}O$$

The reduction of nitrate to nitrogen oxide requires the presence of three parts of vanadium(III) for every one part of nitrate. Therefore, a solution of 0.015 M VCl_3_ is capable of reducing 0.005 M NO_3_^−^, which corresponds to a sample concentration of 140 mg L^−1^ NO_3_^−^. This concentration can be injected into the system and completely reduced if we consider that the mobile phase and reducing agents are mixed in a 1:1 ratio before they enter the reduction oven. The reduction of nitrate with vanadium(III) requires strongly acidic conditions at or below pH 1 [7], which was provided by the 0.3 M HCl solution in which the vanadium(III) chloride was solved. For determination of conversion rates and selection of the acid, see Supporting Information S1 and S2. The solution was prepared with degassed Milli‐Q water to avoid oxidation with dissolved oxygen and stored in a refrigerator for up to 1 week. The eluent was prepared by diluting sulfuric acid (95%–97%, Merck, Darmstadt, Germany) to a concentration of 0.005 M H_2_SO_4_ or to a concentration required for the experiment.

All solutions were subjected to further purification under vacuum by a membrane pump (Vacuubrand GmbH & Co., Wertheim, Germany) and in an ultrasonic bath (Sonorex RK 100 Bandelin Electronic, Berlin, Germany) for a minimum of 15 min. They were continuously flushed with a small flow of helium (5.0) (Air Liquide, Oberhausen, Germany) throughout their use. The isotopic reference materials USGS 32, 34, and 35 (IVA Analysentechnik GmbH & Co. KG, Meerbusch, Germany) and nitrate in‐house standards KNO_3_ and NaNO_3_ (> 99%, Merck, Darmstadt, Germany) were measured on an Isoprime100 Elemental Analyzer (Elementar Analysensysteme GmbH, Langenselbold, Germany) for referencing purposes. The standard solutions typically contained 50 mg L^−1^ N‐NO_3_^−^ (nitrate‐N). One standard dilution series was prepared with concentrations of 10, 25, 50, 75, and 100 mg L^−1^ N‐NO_3_^−^ to assess the concentration dependence of the system. For the determination of standard bulk isotope ratios measured by EA‐IRMS, see Supporting Information S4.

### 2.3 Experimental Setup

A scheme of the modified system is depicted in Figure 1. The modified system based on the commercially available LC IsoLink interface (Thermo Fisher Scientific, Bremen, Germany) coupled to a DELTA V Advantage IRMS (Thermo Fisher Scientific, Bremen, Germany) for continuous flow applications. Eluents and reducing agents were pumped with two separate HPLC pumps (LPG‐3400 SD and HPG‐3200 SD, Thermo Fisher Scientific, Bremen, Germany). The flow rates for the eluent and reducing agents were both set to 200 μL min^−1^. Nitrate injections were performed by an HTC PAL autosampler (CTC Analytics AG, Zwingen, Switzerland) with different sizes of PEEK sample loops (5, 10, and 20 μL). The separation of nitrate from the injection peak and other potential matrix effects was achieved with a Hypercarb PGC column (2.1 × 100 mm, 3 μm, Thermo Fisher Scientific GmbH, Bremen, Germany) and an eluent of 0.005 M H_2_SO_4_. Furthermore, the column temperature was elevated to 80°C and maintained by an HT‐HPLC 200 column oven (Scientific Instruments Manufacturer GmbH, Oberhausen, Germany) in order to enhance control over retention times and peak shape. The reducing agent was introduced to the eluent via a corrosion‐free, low dead volume mixing chamber integrated into the commercial LC IsoLink interface and pumped into a heated deactivated fused silica capillary (i.d. 0.32 mm; length 8 m, BGB Analytik, Böckten, Switzerland) for reduction. The dimensions of the fused silica capillary result in a reactor volume of approximately 0.64 mL and a residence time of the analytes that is longer than 1.5 min, with a combined flow rate of 0.4 mL min^−1^. Heating was facilitated by a small self‐made temperature‐controlled GC oven, which was controlled by an Eurotherm 2216e microprocessor (Schneider Electric Systems, Limburg, Germany) (see Part 2 and Figure S1). The mobile phase was cooled after reduction by immersing the capillary (20 cm length, 0.32 i.d., BGB Analytik, Böckten, Switzerland) in a water bath at RT (23°C) before entering the gas separation unit of the IsoLink LC‐IRMS interface. A small inline filter made of 4.9 mm diameter, 10 μm pore size, PEEK encased (IVA Analysentechnik & GmbH, Meerbusch, Germany) was installed before the gas separation unit to shield the three membranes from potential nonsoluble particles. The analytes were extracted and transported by a helium stream (Helium 5.0, Air Liquide, Oberhausen, Germany) of approximately 1–2 mL min^−1^ through the two Nafion membranes of the interface and into two subsequent heated copper reactors. Each reactor consisted of four individual copper wires (length 28 cm; o.d. 0.125 mm) twisted and inserted into a heated ceramic tube (length 320 mm; i.d. 0.5 mm) (both IVA Analytik, Meerbusch, Germany). This tube was inserted into a custom‐made oven, which was controlled by a JUMO iTRON 16 (JUMO GmbH & Co. KG, Fulda, Germany) microprocessors and held at 650°C. The heated copper was used to reduce gaseous nitrogen oxide species to elemental nitrogen in accordance with the following equation:

(2) $$y\;\mathrm{Cu}+N_{x}O_{y}\to y\;\mathrm{CuO}+\frac{x}{2}N_{2}$$

The regeneration of the copper reactors was achieved by a stream of 3% H_2_ in He (Crystal Mixture, Air Liquide Düsseldorf, Germany) at 3–4 mL min^−1^.

### 2.4 Test of Reference Gas Stability

The intensity of the reference gas peaks was controlled by adjusting the gas pressure of the reference gas within the system using the pressure regulator of the interface. At the same time, the sample inlet split on the LC IsoLink interface was opened, whereas a 0.05 M H_2_SO_4_ eluent and 0.015 M VCl_3_ in 0.3 M HCl reducing agent were pumped at a rate of 200 mL min^−1^ each.

### 2.5 Enrichment Experiment

The Rotbach River is a minor tributary of the Rhine in western Germany (51.5724° N, 6.6871° E). The enrichment with heavy nitrogen (^15^N) was carried out by using isotopically enriched ^15^NH_4_Cl (Silantes, minimum 99 atom% ^15^N purity). It was diluted in 40 L distilled water for each enrichment experiment. This tracer solution was released via a Duran glass Mariotte's bottle (Schott, Mainz, Germany), which assured a consistent release of the solution independent of the hydrostatic pressure within the bottle. In May and June 2021, a bottle of the labeled material was exposed to the river in order to allow a constant portion to leach into the stream over the course of 6 weeks. Water samples were collected at designated locations on a weekly basis at distances of 50, 100, 200, 300, 500, 750, 1000, 1500, and 2000 m downstream from the administration point. Additionally, one sample was collected 50 m upstream as a reference. The samples were subsequently frozen and stored until further analysis. Given that the modified LC IsoLink interface was not suitable for direct injection and measurement of nitrate from water samples, we proceeded to preconcentrate the water samples after thawing in a vacuum evaporator. This involved evaporating 50 mL of each water sample at 60°C and under 50 mbar to approximately 1 mL, thereby enriching the samples by a factor of 50. Separate spectroscopic measurements of ammonium, nitrite, and nitrate (Table S6) demonstrated that the nitrate concentration in the river was approximately 7 mg L^−1^, which corresponds to 1.58 mg L^−1^ N. Enriching the nitrate concentration by vacuum evaporation 50‐fold would result in a concentration of ~80 mg L^−1^ N, which should fall within the previously determined measurement range of the modified interface. Although the system still exhibits a nonlinear shift in δ^15^N values and peak areas, this shift should not impede the ability to detect enriched nitrogen isotope ratios. We proceeded to evaporate and measure a 1:50 diluted sample of the in‐house standard at an initial concentration of 50 mg L^−1^ N. This was done to ascertain the impact of isotope fractionation effects during evaporation. Following evaporation, all samples were filtered through 0.2 μm PTFE filters (Fisher Scientific, Schwerte, Germany), and 10 μL of the resulting sample volume was directly injected into the system.

## 3 Results and Discussion

### 3.1 Stability and Linearity of IRMS Under Measurement Conditions

In order to assess the precision of the IRMS system for nitrogen isotope ratios under measurement conditions, 10 consecutive reference gas peaks were injected with constant (stability) and increasing (linearity) signal intensities. Table S2 presents the average (Avg) and standard deviation (SD) of *δ* ^15^N values from 10 reference gas peaks (denoted by *w*). These values were compared to linearity measurements without background signals, whereby the sample open split was turned off (denoted by *wo*). The SD of linearity measurements of nitrogen isotope ratios without any background signals under ideal IRMS conditions was 0.06‰ and increased to 0.41‰ conducting the same measurements with an active sample open split. The SD of stability measurements with an active sample open split (*w*) was 0.37‰. The main cause for the increasing SD of measurements with an active open split were outliers (peak no. 6 for linearity and peak no. 4 for stability measurements, marked with * in Table S2) throughout the complete series of 10 reference gas injections. The cause for these outliers were small shifts in the *m/z* 29/28 ratios (see Figure S2) occasionally appearing throughout chromatograms. The shifts tend to be 1 min long, and, due to the nature of the interface to produce wide peaks, they might originate from the liquid phase of the system, possibly due to piston movement of the HPLC pumps. We took great care to manually check and avoid measurements where one of these shifts occurred under peaks of interest. Despite these interferences, the system performs sufficiently robust and is able to measure nitrogen isotope ratios of elemental nitrogen with a precision better than 0.5‰ over a peak area range of 4.6–21.3 Vs and possibly more precise if background shifts are avoided.

### 3.2 Separation of Nitrate and Nitrite From Injection Peak

**FIGURE 2:**
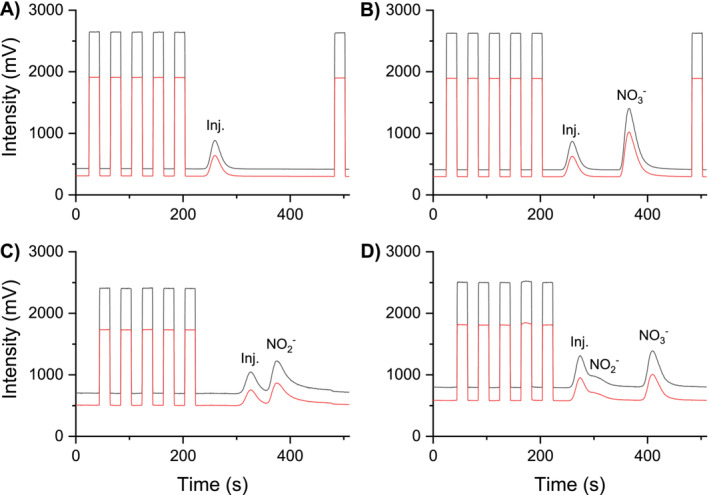
Chromatograms of blank (A), nitrate (B), nitrite (C), and a mix of both nitrate/nitrate (D) injections into the modified LC‐IRMS interface for the measurement of nitrogen isotope ratios. Blank injections produce a measurable nitrogen peak (A).

**FIGURE 3:**
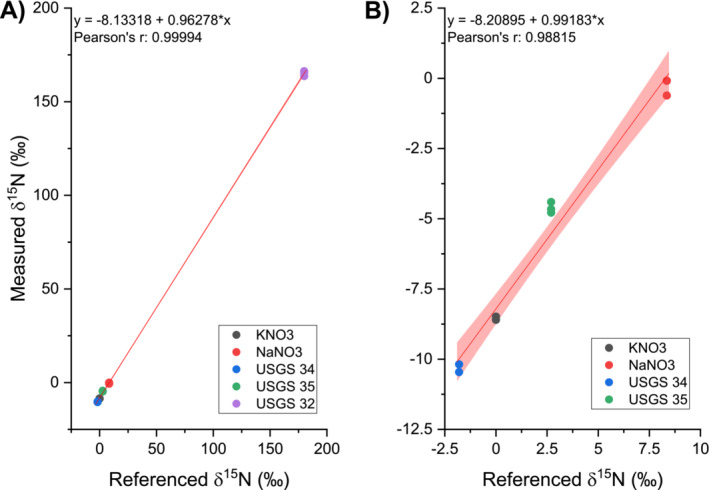
Differences between measured and referenced *δ* ^15^N values of reference materials and in‐house standards were in good agreement. Because USGS 32 is an enriched reference material (A), it was removed for the linear regression (B) to decrease the range of *δ* ^15^N values to more natural abundance levels. Red line indicates linear regression curve and red area gives the 95% confidence interval. Samples were injected in triplicates.

**FIGURE 4:**
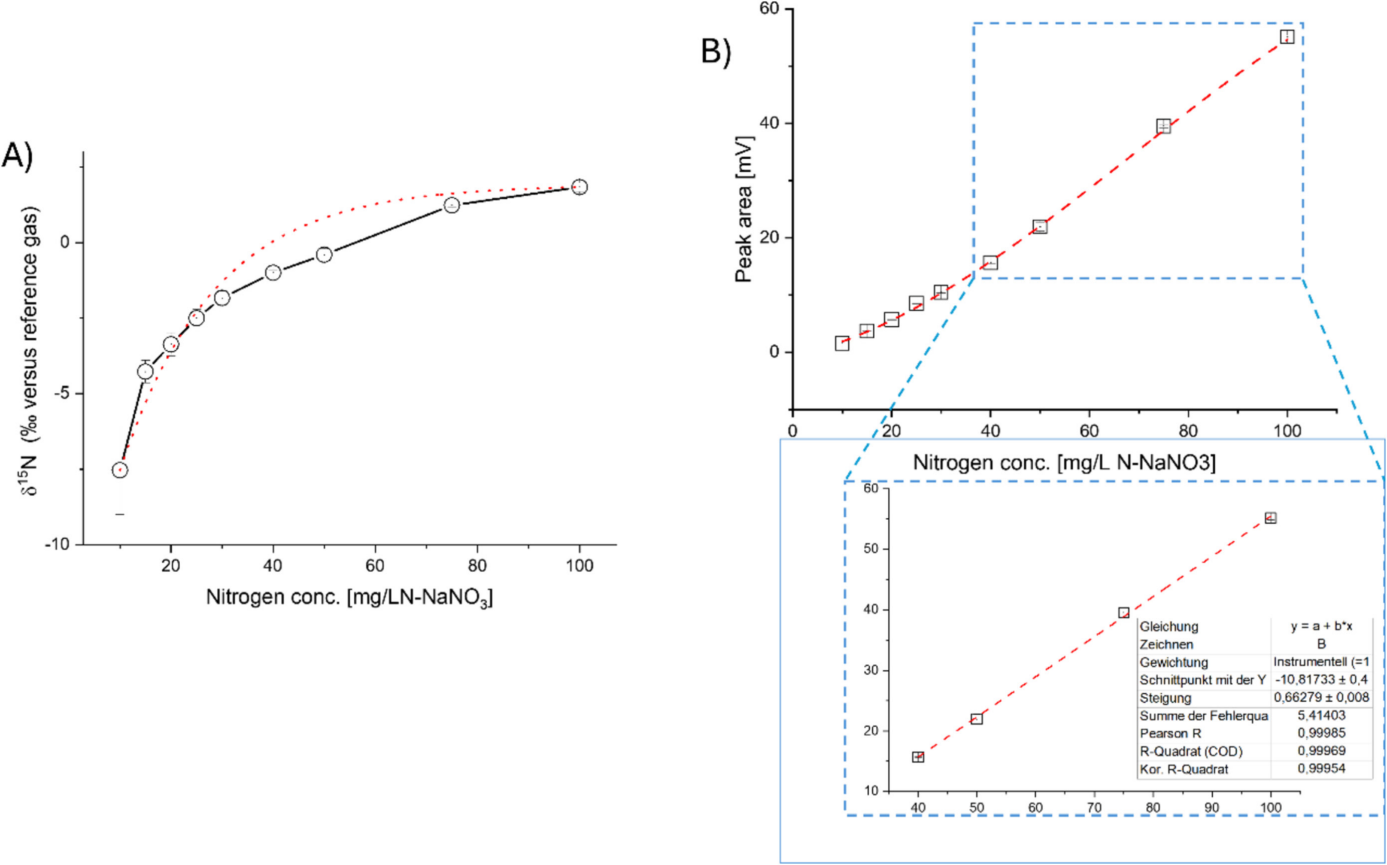
Injection of nitrate in different concentrations leads to a shift of measured *δ* ^15^N values (A) and a nonlinear increase in peak areas (B). 10 μL NaNO_3_ solutions from 10 to 100 mgL^−1^ N‐NO_3_ were injected in triplicate and the δ^15^N values (‰) and peak area (Vs) measured. Peak integration was done by ISODAT software with the default settings of 0.2 and 0.4 mV s^−1^ start and end slope detection and an individual background detection algorithm with a 5 s history. The increase in peak area over the concentration range is best described by a second polynomial equation with R‐square of 0.99812. A linear behavior from 40 mg L^−1^ with a Pearson coefficient *r* of 0.99985 (see lower panel of (B)).

**FIGURE 5:**
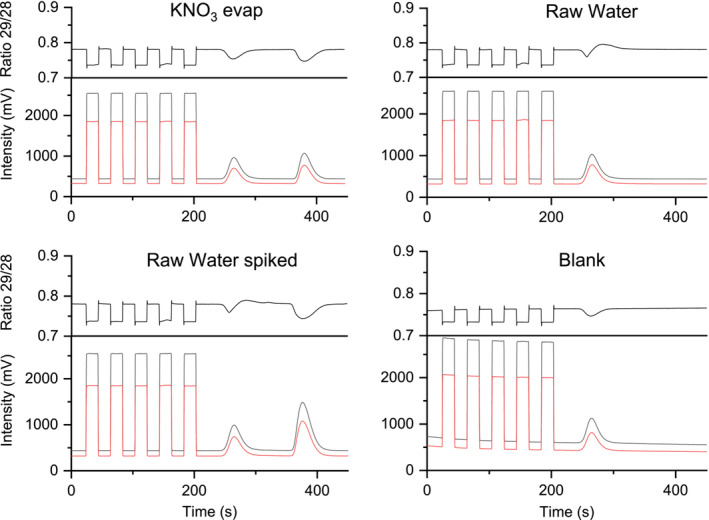
Chromatograms show measurements of evaporated KNO_3_ standard solutions (top left), river water (top right), river water spiked with a nitrate standard (bottom left), and a blank sample of evaporated Milli‐Q water (bottom right). Evaporation overnight in a vacuum evaporator does not influence the *m/z* 29/28 ratio of either the nitrate peak (around 380 s) or injection peaks.

Blank injections into the modified interface produced significant nitrogen peaks (Figure 2A), which also occurred with no reducing agents in the mobile phase and when the reactors were at room temperature. The measured *δ* ^15^N signatures of these peaks were between +2 and +8‰ against the nitrogen reference gas, which was set to 0. We therefore assumed that these blank peaks were either produced by dissolved elemental nitrogen in the sample or by switching the valve position of the six‐port valve from the autosampler. Switching the valves might have introduced small amounts of elemental nitrogen from the surrounding air into the eluent flow of the system. Separation of nitrate from the injection peak and nitrite was therefore crucial to avoid interferences and was achieved by a porous graphitic carbon (PGC) HPLC column (Hypercarb 100 × 2.1 mm, 5 μm, Thermo Scientific). We used an elevated column temperatures at around 80°C and diluted sulfuric acid as anionic competitor to elute nitrate from the column. Without an anionic competitor, nitrate would be totally retained on the column. Sodium sulfate was used by Takeuchi et al. to separate inorganic anions including nitrate and nitrite on a PGC column [8], but we decided to use diluted sulfuric acid (0.005 M H_2_SO_4_) instead to keep the pH as low as possible for subsequent reduction (pH ~1). However, due to the broad peak widths produced in our modified interface and the LC‐IRMS interface in general, efficient separation of nitrite and nitrate from the injection peak was not possible simultaneously (Figure 2B). Here, the HPLC flow was set to 200 μL min^−1^ for both the eluent and reducing agent (0.03 M VCl_3_ in 0.3 M HCl) and reactor column temperature was held at 55°C. As the focus of this work was the measurement of nitrogen isotope ratios from nitrate, separation of nitrate from both blank and nitrite peaks was considered sufficient. Injecting a mixture of nitrate and nitrite (50 mg L^−1^ N each, 5 μL injection volume) under these flow conditions (Figure 2D) showed that nitrite was not fully separated from the injection peak. Reducing the eluent flow from 200 to 150 μL min^−1^ and increasing the column temperature to 80°C (Figure 2C) almost separated nitrite (100 mg L^−1^ N, 5 μL injection volume) from the injection peak. Note that the injection volume was reduced from initially 10 μL (Figure 2A,B) to 5 μL (Figure 2C,D) to reduce the load of nitrogen oxides on the copper reactor. The concentration of sulfate in the eluent as well as flow rates and column temperatures can be used to influence the retention time of nitrate. Testing different column parameters and materials might result in an improved performance of the system to separate both nitrate and nitrite from the injection peak.

### 3.3 Repeated Nitrate Measurements and Reactor Performance

To evaluate the ability of the copper reactor to reduce the high loads of nitrogen oxides coming from the modified LC‐IRMS interface in addition to the oxygen loads coming from the eluent, we consecutively injected 5 μL of a 50 mg L^−1^ N solution of the USGS 34 international reference material on 3 following days. The copper reactor was reduced overnight in between measurement days with a 2 mL min^−1^ flow of 3 vol‐% H_2_ in He gas. The measured *δ* ^15^N values were constant for four to six injections, after which they started to decrease until the 20th to 25th injection (see Figure S3 and Table S3). This indicated that the reactor performance is stable for the first few injections but quickly declines afterwards. Overnight regeneration of the reactor increased the measured *δ* ^15^N values on the next day, but not to the same values previously observed. This showed that though regenerating the reactor is feasible, there might still be daily differences in reactor or system performance. Thus, frequent referencing through international or in‐house standards, as typically required and practiced in CSIA, minimized the daily influence on the performance. Measuring nitrogen isotope ratios from nitrate with high precision throughout a day or for prolonged sample runs might therefore require a combination of two copper reactors in parallel, where one reactor is used for one or several injections of nitrate containing samples and the other is regenerated with a separate line of hydrogen gas as described in the literature [9].

### 3.4 Measuring Different Nitrogen Reference Materials With a Wide Range of Isotopic Signatures

Two international nitrate reference materials with certified nitrogen isotope ratios on natural abundance levels (USGS 34 and USGS 35) and one material enriched in its nitrogen isotope ratio (USGS 32) were utilized in this study. Additionally, two in‐house nitrate standards (KNO_3_ and NaNO_3_) were calibrated on an EA‐IRMS system with the three USGS reference materials. The referenced values of all five standard and reference materials are summarized in Table S1. We injected 10 μL of a nitrate solution containing 50 mg L^−1^ N‐NO_3_^−^ of each material in triplicate and plotted the measured *δ* ^15^N values with all standard and reference materials (Figure 3A) and with materials on natural abundance levels (Figure 3B). The y‐intercept of a linear regression curve between referenced and measured *δ* ^15^N values shows that measured *δ* ^15^N values are −8.1‰ and −8.2‰ lower than the referenced values, but a slope of 0.96 and 0.99 indicates that the differences between measured values of different materials are in good agreement to the differences of referenced values. As mentioned earlier, we occasionally observed small shifts in the *m/z* 29/28 ratio during measurements, which resulted in higher measured *δ* ^15^N values if the shifts overlapped with either a reference gas peak or a chromatographic peak and caused the observed difference. The reported values were produced throughout continuous measurements on two individual days, and the copper reactors were regenerated overnight. The results are therefore also subject to isotope shifts over extended measurement periods and to different copper reactor performance on individual sampling days. Addressing these issues might therefore further improve the precision and linearity of the regression. However, the reported values already show that the measurement of nitrogen isotope ratios from nitrate with the current setup was feasible on both natural abundance and enriched levels.

### 3.5 Measuring Different Nitrate Concentrations

To test the influence of nitrate concentration on nitrogen isotope ratios of nitrate, we prepared and measured a dilution series of NaNO_3_ in‐house standards. 10 μL injections of different N‐NaNO_3_ solution concentrations were done in triplicate and the measured *δ* ^15^N values and peak areas were plotted against the nitrogen concentration (Figure 4A,B). δ^15^N values increase non‐linear from −7.5 ± 1.4‰ to +1.8 ± 0.1‰ over a range of 10–100 mgL^−1^ N‐NaNO_3_, whereas peak areas simultaneously increase nonlinear from 1.6 ± 0.0 Vs to 55.1 ± 0.3Vs and are best described by a second‐order polynomial equation with a Pearson coefficient *r* of 0.99812. The measured δ^15^N values of 40 mg L^−1^ N‐NaNO_3_ was 0.4 ± 0.2‰. The lowest injected nitrate concentration of 10 mg L^−1^ N shows a noticeable increase in measurement uncertainty and the largest shift of δ^15^N values. In the magnified lower part of Figure 4B, it is shown that the nitrate concentration plotted against the peak area showed a linear behavior from 40 mg L^−1^ with a Pearson coefficient *r* of 0.99985. These concentrations are about 10–50 times higher than what is usually observed in water samples from areas affected by substantial agricultural activities, which results in nitrate concentrations between 10 and 50 mg L^−1^ N‐NO_3_^−^ [10]. Especially, nitrate concentration in groundwaters or areas with limited agricultural activities were below the currently expected detection limits of the modified interface. As mentioned earlier, increasing the injection volume is a potential way of achieving the necessary analyte amount on the column if chromatographic separation is assured and the column is not overloaded. However, in our current setup, sample enrichment prior to injection was necessary. Such an enrichment could be achieved by evaporation under vacuum (see next section) or by the use of anion exchange columns to preconcentrate nitrate, as described by the ion‐exchange method [11].

### 3.6 Sample Evaporation, Blank Samples, and Raw River Water

In our study, we decided to test and employ sample evaporation to increase the nitrate concentration in real water samples. Injections of 10 μL Milli‐Q water (blank) into the system show blank peaks of dissolved nitrogen gas in the IRMS system after 260 s under the employed conditions, which had *δ* ^15^N values between +4‰ and +10‰ measured against the reference gas. No nitrate peak was visible in blank samples (Figure 5, bottom right). To test if evaporation of a nitrate solution leads to isotope fractionation, we diluted (1:50) standard solutions of 50 mg L^−1^ KNO_3_ and 25 mg L^−1^ NaNO_3_ and evaporated 50 mL down to 1 mL sample volume. Injecting 10 μL of the evaporated standard solutions and measuring the nitrogen isotope ratio showed no significant difference in *δ* ^15^N values compared to non‐evaporated standard samples (Welch's *t*‐tests, *t*_2_ = −2.488, *p* = 0.124 for NaNO_3_ and *t*_2_ = 0.257, *p* = 0.821 for KNO_3_). The reported standard deviations for evaporated standard solutions were noticeable higher (1.7‰ for KNO_3_ and 1.2‰ for NaNO_3_) compared to normal standard solutions (0.2‰ for both KNO_3_ and NaNO_3_). Injecting raw river water from an isotopic enrichment experiment with ^15^NH_4_Cl, which was not evaporated to preconcentrate nitrate, also showed an injection peak after 265 s and no measurable nitrate peak (Figure 5, top right). The injection peak from ^15^N‐enriched raw water samples, however, exhibited a visible positive shift in *m/z* 29/28 ratios leading to an increased δ^15^N value of +91.0 ± 2.5‰. The observed positive shift appeared not immediately with the beginning of the peak, where signal intensities start to rise, but shortly afterwards before the peak intensity reached its maximum. Spiking a raw water sample with nitrate in‐house standards resulted in a nitrate peak after 376.2 s, whereas the positive isotope shift during the injection peak remained. The injection peaks of evaporated standard solutions further did not exhibit the unusual positive shift of the *m/z* 29/28 ratio observed in ^15^N‐enriched raw water samples, and the δ^15^N values of the injection peaks remained under 10 ‰ and comparable to blank or regular standard solutions.

### 3.7 River Samples From an Enriched Ammonium Chloride Administration Experiment

**FIGURE 6:**
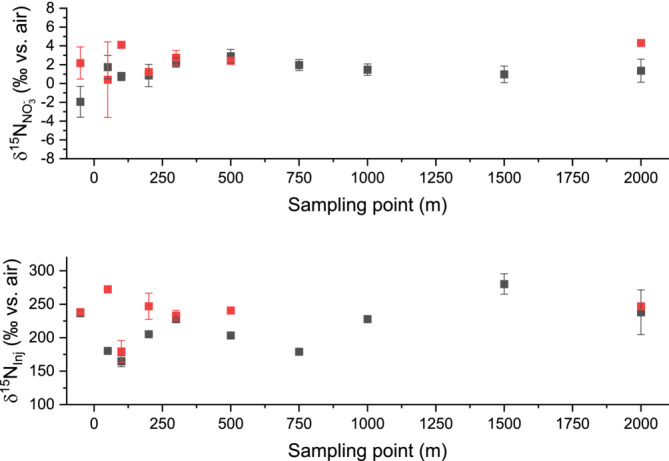
Nitrogen isotope ratios (‰ vs. air) of nitrate (NO_3_^−^, top) and the injection peak (Inj., bottom) from evaporated water samples of the river Rotbach after 1 week (gray boxed) and 2 weeks (red boxes) of introducing isotopically enriched ammonium chloride. Samples were taken up to 2000 m downstream of the administration point and one sample 50 m upstream as a reference.

The measured nitrate concentrations in the river Rotbach were between 6 and 8 mg L^−1^ (see Table S6), and the resulting nitrogen concentrations were around 1.2 mg L^−1^ N‐NO_3_^−^, which could not be measured directly due to low sensitivity. Thus, we measured the nitrogen isotope ratio of N‐NO_3_^−^ from concentrated river water 1 and 2 weeks after administration of isotopically enriched ammonium chloride. In contrast to expectations, *δ* ^15^N values of river nitrate stayed between +0.4 ± 0.4‰ and +4.1 ± 0.3‰ in those 2 weeks, varying over a small range of 3.7 ‰ (Table S4). No trends in δ^15^N values were observed between sampling points or between sampling weeks (see Figure 6). After the second week, the reference point had a δ^15^N value of +2.2 ± 1.7‰, which was well within the observed range of *δ* ^15^N values of nitrate downstream the administration point.

**FIGURE 7:**
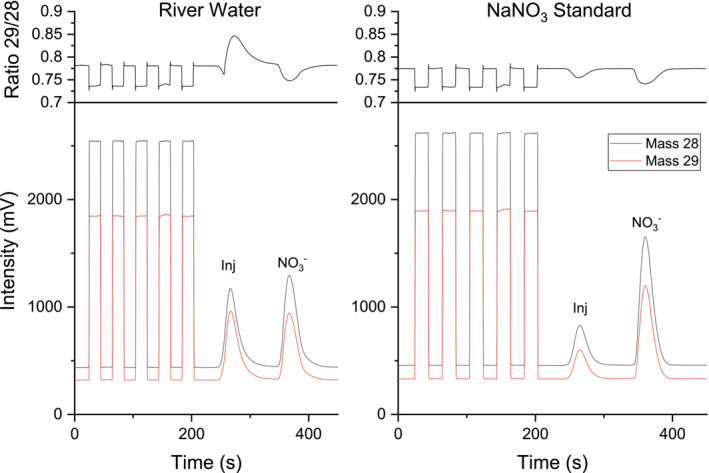
Chromatogram of evaporated river water (left) and NaNO_3_ standard solution at 50 mg L^−1^ N‐NO_3_^−^ (right) showing separation of nitrate from the injection peak. Lines in the bottom section represent signal intensities (mV) of *m/z* 28 (black) and 29 (red) for the determination of *δ* ^15^N values by the IRMS. The upper sections show the ratio of *m/z* 29/28 intensities and reveal an unusual swing in the injection peak for the river water sample, which is typically not observed.

The *δ* ^15^N values of the injection peaks for all samples during that time were much higher and ranged between +164.8 ± 8.1‰ and +272.4 ± 1.2‰, but also with no observable trend either between sampling points or weeks. The chromatograms of evaporated river water showed highly enriched compound eluting from the column right after the start of the injection peak (see Figure 7), as the *m/z* 29/28 ratio showed a strong positive shift shortly after the beginning of the chromatographic peak, which was comparable to what we also observed in non‐evaporated water samples.

The isotope swing observed in raw water samples during the injection peak represents an interesting result, leading to highly increased *δ* ^15^N values of the injection peak around ~90. This indicates that either the atmospheric nitrogen in the river water is highly enriched in ^15^N or some enriched nitrogen oxide species are present in the sample, which are subsequently reduced and measured as elemental nitrogen in the IRMS. The first possibility might be unlikely, because the isotope swing does not completely overlap with the nitrogen injection peak and has a small offset before the positive shift in *m/z* 29/28 ratio occurs. This would indicate that the observed isotope swing during the injection peak of raw river water is caused by partial coelution of isotopically enriched nitrite. The abundance of nitrite in river water is two orders of magnitude lower compared to nitrate, which means that the isotope composition of nitrite in the water sample would be much higher than the measured *δ* ^15^N value of ~90‰, as it coelutes with the much more abundant nitrogen injection peak and typically has a *δ* ^15^N value of ~5‰. This is supported by measurements of evaporated river water, where the δ^15^N value of the injection peak is even higher and reaches over +200‰, which would be a direct result of concentration of nitrite alongside nitrate in the sample material. In the case of the administration study, our results suggest that concentration of nitrate in water samples via evaporation under vacuum is feasible without isotope fractionation and the measured values of evaporated as well as nonevaporated nitrate standards are statistically not distinguishable (Table S5).

Another more likely possibility is that the high delta values of the injection peak for the river samples are caused by carbon monoxide that is probably formed from dissolved organic carbon (DOC). Carbon has a delta value of about +500 if expressed on the δ^15^N scale, so even relatively small contributions of CO could explain these high delta values.

It turned out that for future applications, some adjustments of the system are necessary. The most important improvements are a better chromatographic separation of the nitrite from the injection peak so that a direct measurement of nitrite and nitrate would be possible. The second improvement would be the construction of a reduction furnace with higher capacity and switchable Cu regeneration to reach stable conditions for a prolonged measurement period. The third improvement concerns a better separation of carbon dioxide. Instead of operating the water trap with acetone and dry ice, it can also be operated with an Ascarite trap and an additional liquid nitrogen bath to remove both water and carbon dioxide from the gas phase, which can interfere in the ion source through isobaric interferences of CO for the measurement of elemental nitrogen.

## 4 Conclusions

In this study, we presented a modified LC‐IRMS interface for the measurement of nitrogen isotope ratios of nitrate from aqueous solutions. Chromatographic separation of nitrate from other nitrogen containing compounds and consecutive reduction to elemental nitrogen was achieved and nitrogen isotope ratios were measured from injections from up to 20 μL aqueous samples. We accomplished chromatographic separation of nitrate from blank peaks to increase accuracy and decrease matrix interferences.

Under current conditions, measurements of in‐house standards and reference materials showed that the linearity of measured *δ* ^15^N values were in good agreement with both literature and referenced *δ* ^15^N values from an EA‐IRMS system. Because of the early state of the modified system, this study can be interpreted as a proof of principle. Major challenges that should be addressed in the future include (i) reducing the nitrogen background from the eluent, (ii) modifying the copper reactors to operate in parallel to enable simultaneous regeneration of oxidized copper wires, and (iii) reducing peak broadening by testing different column materials and optimizing flow conditions both in the liquid and gas phase. (iv) Nitrogen blank contribution may be an issue, and further investigation is needed to ensure that there is no nitrogen blank coeluting with and interfering with the nitrate‐N. In future measurements, this can be done by measuring nitrates of very different isotopic compositions at different concentrations to show that the offset between true and measured values is similarly concentration dependent for nitrates of different isotopic compositions. (v) In addition, positioning of the switching valve heads into a nitrogen free gas atmosphere could reduce the amount of air entering the system. These modifications will likely address the observed issues of low sensitivities as well as drifting isotope ratios over time. A separation and measurement of both nitrite and nitrate with one measurement might be possible if separation with other HPLC columns can be optimized.

## Author Contributions

**Tobias Hesse:** methodology, sampling, formal analysis, investigation, visualization, writing–original draft. **Felix Niemann:** sampling, formal analysis, investigation, review and editing. **Shaista Khaliq:** sampling, formal analysis, review and editing. **Daniel Köster:** conceptualization, methodology. **Julian Enss:** sampling, review and editing. **Christian K. Feld:** sampling, review and editing. **Milen Nachev:** sampling, review and editing. **Klaus Kerpen: i**nvestigation. **Maik A. Jochmann:** conceptualization, methodology, writing–review and editing, supervision. **Torsten C. Schmidt:** conceptualization, methodology, writing–review and editing, supervision.

## Conflicts of Interest

The authors declare no conflicts of interest.

## Supporting information


**Figure S1** Picture of the self‐made GC reactor for the on‐line reduction of nitrate into gaseous nitrogen oxides.
**Table S1** Details about all available nitrate standard materials for this study.
**Figure S2** Injections of 10 reference gas peaks with open sample split and increasing gas pressure. The upper graph shows the measured *m/z* 29/28 ratio and the bottom graph the signal intensities/mV) over time (s). The average background *m/z* 29/28 ratio is indicated by a horizontal green line and a shift in *m/z* 29/28 ratios under the sixth peak is marked as a red area, resulting in increased *δ* ^15^N values of this reference gas peak in Table S2.
**Table S2** Average and SD of linearity and stability tests using ten injections of reference gas peaks (*w*) and (*wo*) background signals. Tests performed with background signals show occasional outliers (marked *) and originate from small shifts in the *m/z* 29/28 ratio throughout chromatograms.
**Figure S3** Consecutive injections of 5 μL USGS34 (50 mgL^−1^ N‐NO_3_^−^) into the modified interface. The copper oven was regenerated overnight with a 2–4 mL min^−1^ flow of 3 vol‐% H_2_ in He in between days. The measured *δ* ^15^N values are constant for the first four to six injections on each day, after which they gradually decrease with each further injection. Regeneration of the copper wires overnight increases the measured *δ* ^15^N values on the next day.
**Table S3:** Referenced and measured *δ* ^15^N values of in‐house standards and international reference materials. Samples were injected in triplicate with 10 μL injection volume and a concentration of 50 mgL^−1^ N‐NO_3_^−^. Although the first injection of USGS 34 was statistically not an outlier according to Grubbs tests with a 95% confidence level due to the low sample size, we removed this value from further analysis because it had an abnormal variation in comparison to the variance of the other standard and reference materials.
**Table S4** *δ* ^15^N measurements of Blank samples, raw and spiked river water and standards of a 50 mgL^−1^ N‐NO_3_^−^ solution of KNO_3_ and 25 mgL^−1^ N‐NO_3_^−^ solution of NaNO_3_ compared to solutions which were diluted 1:50 and then evaporated as described in materials and methods. Evaporation of diluted samples of standard materials does not lead to significant differences in *δ* ^15^N values compared to the raw material, but higher SDs were observed for samples which were evaporated. Since homogeneity of variance was violated, we used two‐sided t‐tests with Welch correction on a 0.05 significance level.
**Table S5** Nitrogen isotope ratios (Avg in ‰ vs. Air) and standard deviations (SD) from evaporated water samples for nitrate (NO_3_^−^) and the injection peak (Inj) on two sampling days in May 2021.
**Table S6** Photometric determination of nitrite, nitrate and ammonium on May 25^th^, 2022, in the river Rotbach on sampling points up to 2000 m downstream from the administration of ^15^N‐enriched NH_4_Cl.

## Data Availability

The data supporting the results of this study are available in the manuscript and a supporting information file. In addition, data are available from the corresponding author upon reasonable request.

## References

[1] X. H. Xia , Z. F. Yang , G. H. Huang , X. Q. Zhang , H. Yu , and X. Rong , “Nitrification in Natural Waters With High Suspended‐Solid Content‐‐A Study for the Yellow River,” Chemosphere 57, no. 8 (2004): 1017–1029, 10.1016/j.chemosphere.2004.08.027. 15488592

[2] D. Köster , M. A. Jochmann , H. V. Lutze , and T. C. Schmidt , “Monitoring of the Total Carbon and Nitrogen Balance During the Mineralization of Nitrogen Containing Compounds by Heat Activated Persulfate,” Chemical Engineering Journal 367 (2019): 160–168, 10.1016/j.cej.2019.02.115.

[3] D. Köster , I. M. Sanchez Villalobos , M. A. Jochmann , W. A. Brand , and T. C. Schmidt , “New Concepts for the Determination of Oxidation Efficiencies in Liquid Chromatography‐Isotope Ratio Mass Spectrometry,” Analytical Chemistry 91, no. 8 (2019): 5067–5073, 10.1021/acs.analchem.8b05315. 30892863

[4] W. A. Brand , “High Precision Isotope Ratio Monitoring Techniques in Mass Spectrometry,” Journal of Mass Spectrometry 31, no. 3 (1996): 225–235, 10.1002/(sici)1096-9888(199603)31:3<225::aid-jms319>3.0.co;2-l. 8799274

[5] S. A. Merritt and J. M. Hayes , “Nitrogen Isotopic Analyses by Isotope‐Ratio Monitoring Gas Chromatography/Mass Spectrometry,” Journal of the American Society for Mass Spectrometry 5 (1994): 387–397, 10.1016/1044-0305(94)85054-2. 24222593

[6] C. F. Stange , O. Spott , B. Apelt , and R. W. Russow , “Automated and Rapid Online Determination of 15N Abundance and Concentration of Ammonium, Nitrite, or Nitrate in Aqueous Samples by the SPINMAS Technique,” Isotopes in Environmental and Health Studies 43, no. 3 (2007): 227–236, 10.1080/10256010701550658. 17786668

[7] R. S. Braman and S. A. Hendrix , “Nanogram Nitrite and Nitrate Determination in Environmental and Biological Materials by Vanadium (III) Reduction With Chemiluminescence Detection,” Analytical Chemistry 61, no. 24 (1989): 2715–2718, 10.1021/ac00199a007. 2619057

[8] T. Takeuchi , T. Kojima , and T. Miwa , “Ion Chromatography of Inorganic Anions on Graphitic Carbon as the Stationary Phase,” Journal of High Resolution Chromatography 23, no. 10 (2000): 590–594, 10.1002/1521-4168(20001001)23:10<590::AID-JHRC590>3.0.CO;2-C.

[9] E. Hettmann , W. A. Brand , and G. Gleixner , “Improved Isotope Ratio Measurement Performance in Liquid Chromatography/Isotope Ratio Mass Spectrometry by Removing Excess Oxygen,” Rapid Communications in Mass Spectrometry 21, no. 24 (2007): 4135–4141, 10.1002/rcm.3304. 18041012

[10] B. He , S. Kanae , T. Oki , Y. Hirabayashi , Y. Yamashiki , and K. Takara , “Assessment of Global Nitrogen Pollution in Rivers Using an Integrated Biogeochemical Modeling Framework,” Water Research 45, no. 8 (2011): 2573–2586, 10.1016/j.watres.2011.02.011. 21402394

[11] S. R. Silva , C. Kendall , D. H. Wilkison , A. C. Ziegler , and C. C. Y. Chang , “A New Method for Collection of Nitrate From Fresh Water and the Analysis of Nitrogen and Oxygen Isotope Ratios,” Journal of Hydrology 228 (2000): 22–36, 10.1016/S0022-1694(99)00205-X.

