# Supplementary material for: A Proof‐of‐Principle Study for δ 15N Measurements of Aqueous Dissolved Nitrate With a Modified LC‐IRMS Interface
Source: Rapid Commun Mass Spectrom. 2024 Nov 26;39(3):e9950. doi: 10.1002/rcm.9950 (PMC11590410; doi:10.1002/rcm.9950)
Supplement: Supplementary file 1 — Figure S1 Picture of the self‐made GC reactor for the on‐line reduction of nitrate into gaseous nitrogen oxides. Table S1 Details about all available nitrate standard materials for this study. Figure S2 Injections of 10 reference gas peaks with open sample split and increasing gas pressure. The upper graph shows the measured m/z 29/28 ratio and the bottom graph the signal intensities/mV) over time (s). The average background m/z 29/28 ratio is indicated by a horizontal green line and a shift in m/z 29/28 ratios under the sixth peak is marked as a red area, resulting in increased δ 15N values of this reference gas peak in Table S2. Table S2 Average and SD of linearity and stability tests using ten injections of reference gas peaks (w) and (wo) background signals. Tests performed with background signals show occasional outliers (marked *) and originate from small shifts in the m/z 29/28 ratio throughout chromatograms. Figure S3 Consecutive injections of 5 μL USGS34 (50 mgL−1 N‐NO3 −) into the modified interface. The copper oven was regenerated overnight with a 2–4 mL min−1 flow of 3 vol‐% H2 in He in between days. The measured δ 15N values are constant for the first four to six injections on each day, after which they gradually decrease with each further injection. Regeneration of the copper wires overnight increases the measured δ 15N values on the next day. Table S3: Referenced and measured δ 15N values of in‐house standards and international reference materials. Samples were injected in triplicate with 10 μL injection volume and a concentration of 50 mgL−1 N‐NO3 −. Although the first injection of USGS 34 was statistically not an outlier according to Grubbs tests with a 95% confidence level due to the low sample size, we removed this value from further analysis because it had an abnormal variation in comparison to the variance of the other standard and reference materials. Table S4 δ 15N measurements of Blank samples, raw and spiked river water and standards of a 50 [file RCM-39-e9950-s001.docx]

Supporting Information for A proof-of-principle study for *δ*^15^N measurements of aqueous dissolved nitrate with a modified LC-IRMS interface

Tobias Hesse^5^, Felix Niemann^1^, Shaista Khaliq^1^, Daniel Köster^4^, Julian Enss^2,3^**,** Christian K. Feld^2,3^, Milen Nachev^2.3^, Klaus Kerpen^1^, Maik A. Jochmann^1,3^, Torsten C. Schmidt^1,3^

^1^Instrumental Analytical Chemistry, University of Duisburg-Essen, Universitätsstr. 5, 45141 Essen, Germany

^2^ Aquatic Ecology, Faculty of Biology, University of Duisburg-Essen, Universitätsstraße 5, Essen, 45141, Germany

^3^Centre for Water and Environmental Research, University of Duisburg-Essen, Universitätsstr. 5, 45141 Essen, Germany

^4^Institut Für Arbeitsschutz der Deutschen Gesetzlichen Unfallversicherung (IFA), Alte Heerstraße111, 53757 Sankt Augustin, Germany

^4^Landesamt für Natur, Umwelt und Verbraucherschutz NRW, Wuhanstr. 6, 47051 Duisburg, Germany

Content of Supporting Information

[1. Methods employed for isotope analysis of nitrate and nitrite 2](#_Toc175568448)

[2. Determination of the chemical nitrate turnover by ion chromatography 3](#_Toc175568449)

[3. Conversion rates to NO_3_^-^ by using V(III)/H_2_SO_4_ or V(III)/HCl 4](#_Toc175568450)

[4. Validation of nitrate reference materials 5](#_Toc175568451)

[5. System stability tests 7](#_Toc175568452)

[6. Evaporation experiments 10](#_Toc175568453)

[7. Nitrate concentrations of river water 11](#_Toc175568454)

[8. References 11](#_Toc175568455)

# Methods employed for isotope analysis of nitrate and nitrite

Several methods have been developed and used for the stable isotope analysis of nitrate and nitrite. Six of these will be discussed in brief:

(i) The ion-exchange method represents the earliest developed approach and employs an anion-exchange column for the preconcentration of nitrate and nitrite. They are then removed from the column by hydrochloric acid and analyzed as silver nitrate and silver nitrite using an elemental analyzer. ^1^

(ii) The denitrifier method uses bacterial strains with lacking nitrous oxide reductase activity to stop the denitrification of nitrate and nitrite at N_2_O, which is purified and trapped prior to IRMS analysis. ^2^

(iii) The cadmium azide method uses a cadmium sponge to reduce nitrate to nitrite in a first step and to further reduce nitrite to nitrous oxide prior to IRMS analysis. ^3^

(iv) Another method describes the measurement of ^15^N abundances of aqueous nitrate and nitrite by on-line reduction to NO with vanadium or titanium chloride and subsequent measurement with a membrane-inlet quadrupole MS (SPINS/MIMS) ^4^ , based on a continuous-flow mass spectrometry method developed in 1999. ^5^ This method has been improved over the years ^6,7^ and the latest iteration uses an IRMS system instead of a quadrupole MS to measure isotopic ratios directly. ^8,9^ Samples with a nitrogen concentration of 35 µmol L^-1^ N for nitrate can be measured and *δ*^15^N values from standards are reported with an accuracy of less than 0.9 ‰.

(v) Wassenaar, Altabet et al. developed a method based on the reduction to N_2_O by reduction with Ti(III) with subsequent cryogenic purification and detection by IRMS ^10^ or without purification by laser spectroscopy. ^11^ The method provides the simultaneous measurement of *δ*^15^N and *δ*^18^O by IRMS and δ^17^O values by laser spectroscopy.

(vi) Recently, Hilkert et al. showed that stable isotope ratio analysis (SIA) of nitrate is possible using ESI Orbitrap. Nitrogen (*δ*^15^N_AIR_), oxygen *δ*^18^O_VSMOW_, and *δ*^17^O_VSMOW_ isotope ratios can be measured simultaneously with a long-term precision of ≤ 0.4 ‰ for reference material and purified nitrate samples. ^12^

# Determination of the chemical nitrate turnover by ion chromatography

To determine the degree of NO_3_^-^ conversion by V(III) in our system, the column was removed from the flowline, the silica capillary was unscrewed prior to the gas separation unit and eluent with 10 mg L^-1^ N-NO_3_^-^ (nitrate-N) as NaNO_3_ (Riedel-de Haen AG, Seelze, Germany) was used. A tray of 10 mL IC-vials was placed in a H_2_O cooling-bath to catch fractions of the flow. 5 mL of effluent were transferred into a 25 mL volumetric flask and filled up with 31.25 mM NaOH (Bernd Kraft GmbH, Duisburg, Germany). This step served three purposes at the same time. The required pH-range for the IC column is 2-12 which made a neutralization step of the acidic flow fractions (< pH 1) necessary. The 1:5 dilution prevented an overloading the IC column with sulphate (SO_4_^-^) and Cl^-^ which were present in large quantities in the matrix. And lastly, the neutralization step ensured that no more NO_3_^-^ is being reduced. A color change of blue/green to yellow after neutralization even indicated the presence of V(V) complexes that are unavailable for further reduction of NO_3_^-^ and NO_2_^-^. An 883 Basic IC Plus (Metrohm, Filderstadt, Germany) was used with a Metrosep A Supp 4 (Metrohm, Filderstadt, Germany) column to separate and quantify the remaining NO_3_^-^.

To estimate if the vanadium concentration in the reducing agent was sufficient to reduce the 10 mg L^-1^ N-NO_3_^-^ in the eluent quantitatively, the stoichiometry is shown in Equation 1 was assumed. Three equivalents of vanadium are necessary to reduce an equivalent of NO_3_^-^. For a vanadium concentration of 20 mM this corresponds to a theoretical maximum reduction capacity of 6.6 mM NO_3_^-^ or 92.4 mg L^-1^ N-NO_3_^-^. The dilution introduced by the mixing of reducing agent and eluent can be neglected because the mixing ratios were kept 1:1 over the course of experiments. It is interesting to note, that earlier experiments with an insufficient concentration of reducing agent showed significant formation of NO_2_^-^.

# Conversion rates to NO_3_^-^ by using V(III)/H_2_SO_4_ or V(III)/HCl

The reduction took place in a self-made GC oven, The oven was designed by Dr. Klaus Kerpen and was built in the mechanical workshop of the University of Duisburg-Essen. In Figure S1B a picture of the oven with the silica capillary.


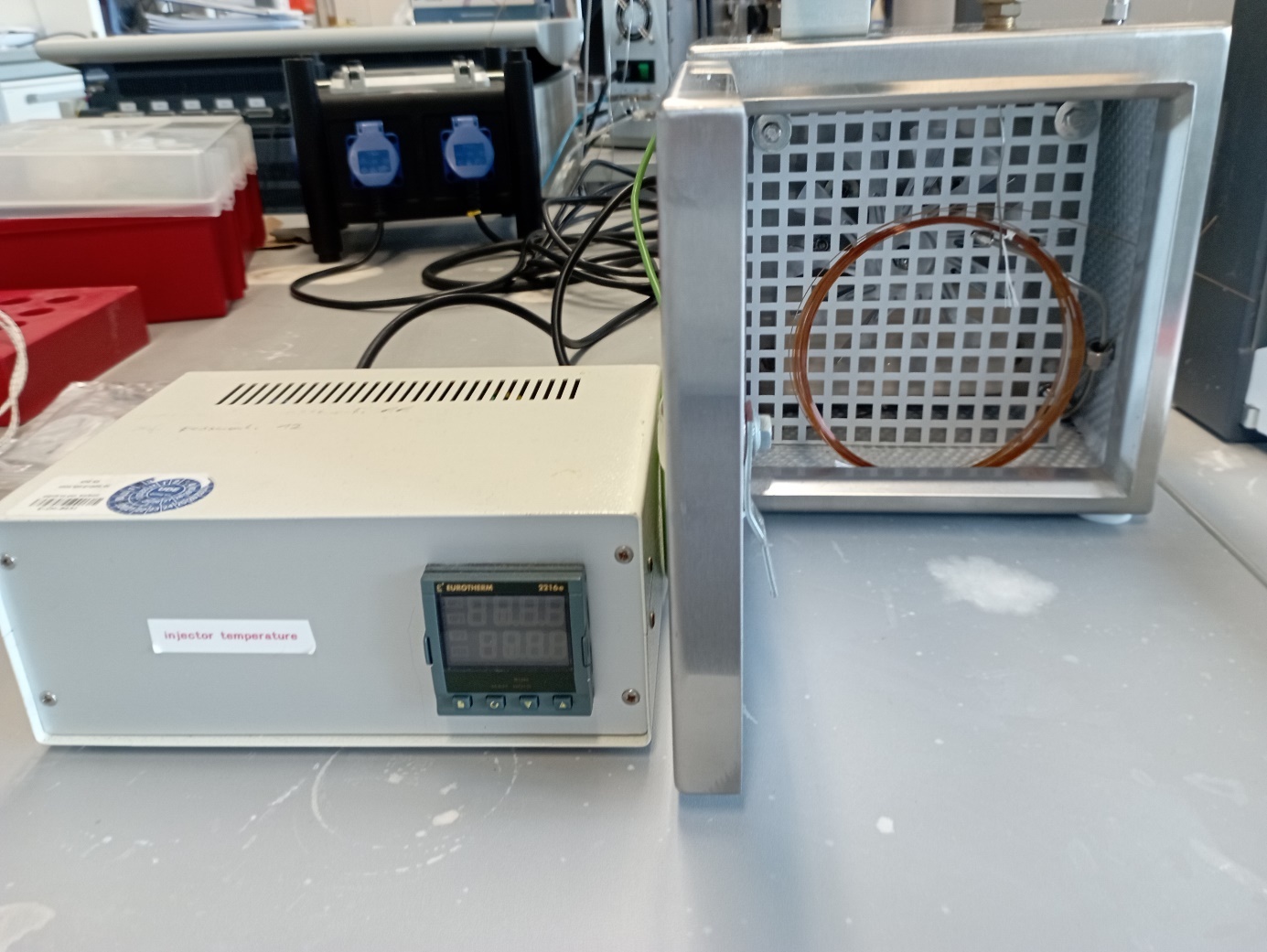


**Figure S1** Picture of the self-made GC reactor for the on-line reduction of nitrate into gaseous nitrogen oxides.

# Validation of nitrate reference materials

The EA-IRMS used to reference the internal laboratory NO_3_^-^ standards was a vario PYRO cube coupled with an IsoPrime100 (both: Elementar Analysensysteme GmbH, Langenselbold, Germany). Two internal NO_3_^-^ laboratory standards and three certified international reference materials were available. The international standards could be used to reference the internal lab-standards by EA-IRMS. All standard materials were weighed into tin cups. Acetanilide served as a control substance to monitor the performance of the instrument throughout the measurement series. NO_3_^-^ stock solutions were prepared by dissolving the respective salts in H_2_O. The internal laboratory standards were prepared and stored in 1 L plastic volumetric flasks with a concentration of 200 mg L^-1^ N-NO_3_^-^. International reference materials were prepared in 25 mL flasks at concentrations of 25 and 50 mg/L N-NO_3_^-^. Details about the standard materials are given in Table S1.

**Table S1** Details about all available nitrate standard materials for this study.


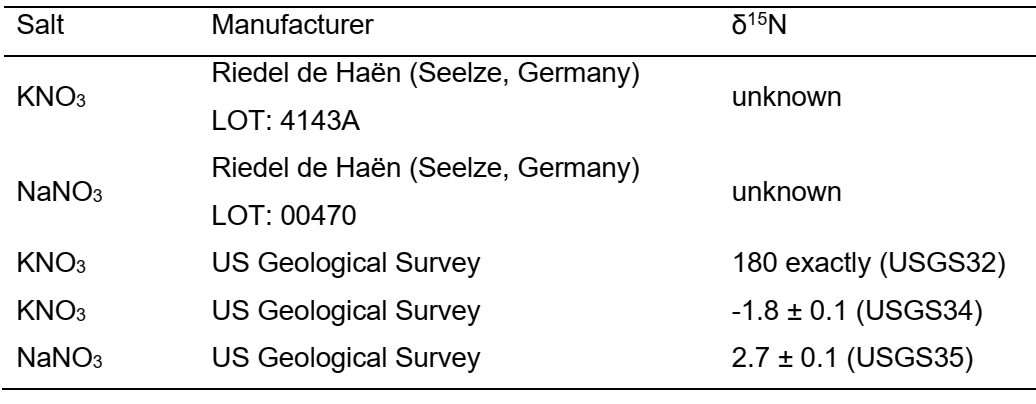


Referencing of isotope data was done by two-point normalization using the in-house nitrate standards KNO_3_ and NaNO_3_, which were already measured and referenced on an EA-IRMS system see before. Their referenced nitrogen isotope ratio of 0.0 ‰ (NaNO_3_) and 8.4 ‰ (NaNO_3_) was used to normalize the measured nitrate isotope ratio of water samples according to the formular

|  | $\delta^{h}E_{c,i-ref}=\frac{\delta^{h}E_{i-ref1}-\delta^{h}E_{i-ref2}}{\delta^{h}E_{m-ref1}-\delta^{h}E_{m-ref2}}*{(\delta}^{h}E_{c}-\delta^{h}E_{m-ref2})+\delta^{h}E_{i-ref2}$ | Eq. S1 |
| --- | --- | --- |

where *δ*^h^E_c_ is the measured isotope ratio of the sample compound and *δ*^h^E_i-ref_ and *δ*^h^E_m-ref_ are the referenced and measured isotope ratios of the reference materials.

# System stability tests


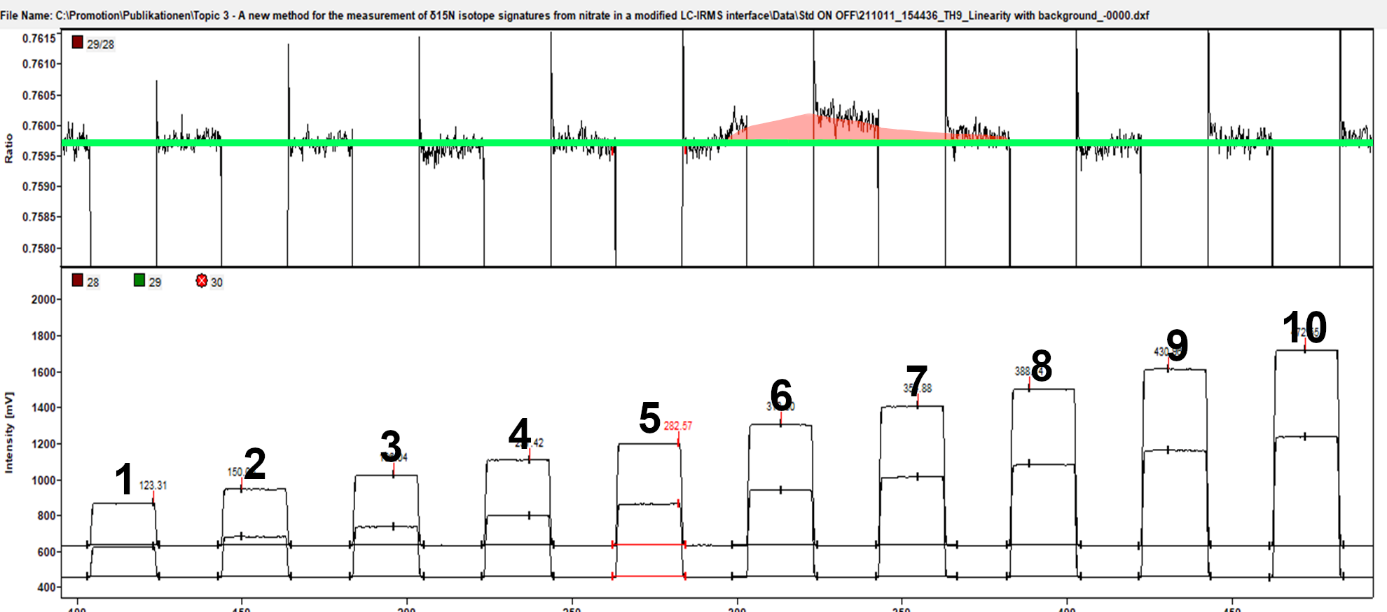


**Figure S2** Injections of 10 reference gas peaks with open sample split and increasing gas pressure. The upper graph shows the measured *m/z* 29/28 ratio and the bottom graph the signal intensities /mV) over time (s). The average background *m/z* 29/28 ratio is indicated by a horizontal green line and a shift in *m/z* 29/28 ratios under the sixth peak is marked as a red area, resulting in increased *δ*^15^N values of this reference gas peak in Table S2.

**Table S2** Average and SD of linearity and stability tests using ten injections of reference gas peaks (*w*) and (*wo*) background signals. Tests performed with background signals show occasional outliers (marked *) and originate from small shifts in the *m/z* 29/28 ratio throughout chromatograms.

| **Peak** | **Linearity (*w*)** | | **Linearity (*wo*)** | | **Stability (*w*)** | |
| --- | --- | --- | --- | --- | --- | --- |
|  | **Area All (Vs)** | **δ^15^N (‰)** | **Area All (Vs)** | **δ^15^N (‰)** | **Area All (Vs)** | **δ^15^N (‰)** |
| 1 | 4.6 | -0.05 | 5.6 | -0.01 | 12.4 | -0.02 |
| 2 | 6.1 | 0.12 | 7.5 | -0.06 | 12.4 | -0.11 |
| 3 | 7.6 | -0.42 | 9.0 | -0.17 | 12.5 | -0.20 |
| 4 | 9.3 | -0.33 | 11.0 | -0.18 | 12.5 | 0.90* |
| 5 | 11.1 | 0.16 | 12.8 | -0.10 | 12.5 | 0.00 |
| 6 | 13.1 | 1.07* | 14.7 | -0.09 | 13.5 | -0.03 |
| 7 | 15.1 | 0.06 | 16.9 | -0.01 | 12.4 | -0.22 |
| 8 | 17.0 | -0.13 | 18.8 | -0.03 | 12.5 | -0.26 |
| 9 | 19.2 | -0.05 | 21.3 | -0.07 | 12.5 | 0.57 |
| 10* | 21.3 | 0.00 | 23.4 | 0.00 | 12.5 | 0.00 |
| **Avg** |  | **0.04** |  | **-0.07** |  | **0.06** |
| **SD** |  | **0.41** |  | **0.06** |  | **0.37** |


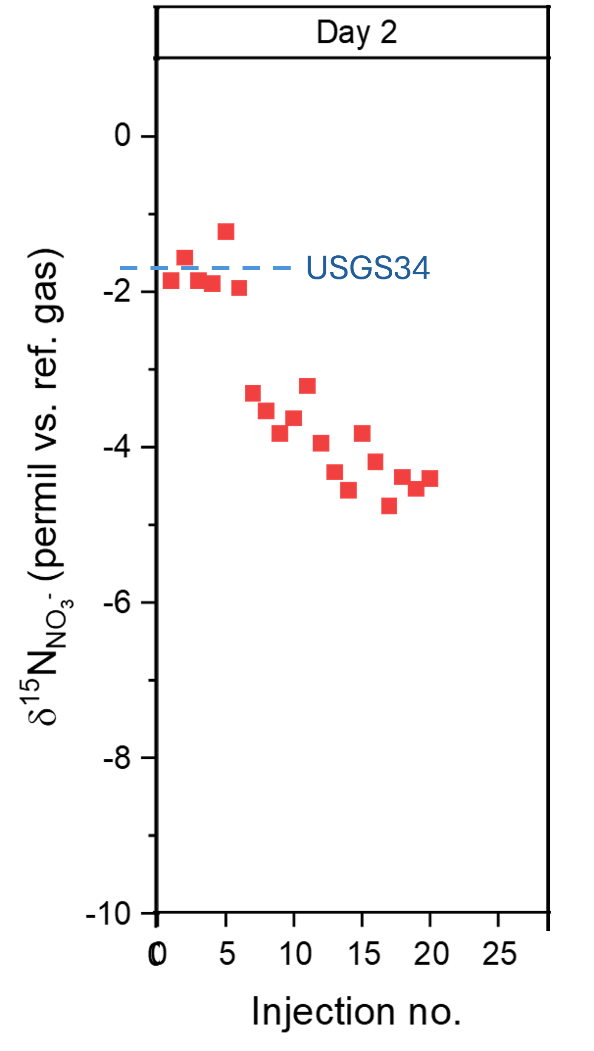


**Figure S3** Consecutive injections of 5 µL USGS34 (50 mgL^-1^ N-NO_3_^-^) into the modified interface. The copper oven was regenerated overnight with a 2 – 4 mL min^-1^ flow of 3 vol-% H_2_ in He in between days. The measured *δ*^15^N values are constant for the first four to six injections on each day, after which they gradually decrease with each further injection. Regeneration of the copper wires overnight increases the measured *δ*^15^N values on the next day.

**Table S3:** Referenced and measured *δ*^15^N values of in-house standards and international reference materials. Samples were injected in triplicate with 10 µL injection volume and a concentration of 50 mgL^-1^ N-NO_3_^-^. Although the first injection of USGS 34 was statistically not an outlier according to Grubbs tests with a 95 % confidence level due to the low sample size, we removed this value from further analysis because it had an abnormal variation in comparison to the variance of the other standard and reference materials.

| **Material** | **Referenced *δ*^15^N** | **Measured *δ*^15^N** |
| --- | --- | --- |
|  | **(‰)** | **(‰)** |
| KNO_3_ | 0.0 | -8.6 |
|  |  | -8.6 |
|  |  | -8.5 |
| NaNO_3_ | 8.4 | -0.6 |
|  |  | -0.1 |
|  |  | -0.1 |
| USGS 35 | 2.7 | -4.4 |
|  |  | -4.8 |
|  |  | -4.7 |
| USGS 32 | 180.0 | 163.6 |
|  |  | 166.3 |
|  |  | 165.5 |
| USGS 34 | -1.8 | -7.6 |
|  |  | -10.2 |
|  |  | -10.5 |

# Evaporation experiments

**Table S4** *δ*^15^N measurements of Blank samples, raw and spiked river water and standards of a 50 mgL^-1^ N-NO_3_^-^ solution of KNO_3_ and 25 mgL^-1^ N-NO_3_^-^ solution of NaNO_3_ compared to solutions which were diluted 1:50 and then evaporated as described in materials and methods. Evaporation of diluted samples of standard materials does not lead to significant differences in *δ*^15^N values compared to the raw material, but higher SDs were observed for samples which were evaporated. Since homogeneity of variance was violated, we used two-sided t-tests with Welch correction on a 0.05 significance level.

| **Sample** | **N** | | **Injection Peak** | | | **Nitrate Peak** | | |
| --- | --- | --- | --- | --- | --- | --- | --- | --- |
|  |  | ***R_t_*** | | ***δ*^15^N** | **SD** | ***R_t_*** | **δ^15^N** | **SD** |
|  |  | (s) | | (‰) | (‰) | (s) | (‰) | (‰) |
| Blank | 3 | 259.3 | | 3.9 | 0.3 | - | - | - |
| KNO_3_ | 3 | 264.4 | | 6.3 | 0.6 | 348.9 | -8.5 | 0.2 |
| KNO_3_ evap. | 3 | 265.3 | | 5.7 | 0.7 | 380.7 | -8.8 | 1.7 |
| NaNO_3_ | 3 | 264.5 | | 5.1 | 0.3 | 349.5 | -3.9 | 0.2 |
| NaNO_3_ evap. | 3 | 265.2 | | 9.4 | 0.2 | 364.2 | -2.1 | 1.2 |
| Raw Water | 2 | 265.3 | | 91.0 | 2.5 | - | - | - |
| Raw Water spiked | 1 | 264.8 | | 68.1 | - | 376.2 | -0.5 | - |

**Table S5** Nitrogen isotope ratios (Avg in ‰ vs. Air) and standard deviations (SD) from evaporated water samples for nitrate (NO_3_^-^) and the injection peak (Inj) on two sampling days in May 2021.

| **Sampling point** | **210518_Inj** | | **210518_NO_3_** | | **210525_Inj** | | **210525_NO_3_** | |
| --- | --- | --- | --- | --- | --- | --- | --- | --- |
|  | Avg | SD | Avg | SD | Avg | SD | Avg | SD |
| -50 | 236.7 | 4.7 | -1.9 | 1.6 | 238.3 | 3.4 | 2.2 | 1.7 |
| 50 | 180.3 | 1.0 | 1.7 | 1.2 | 272.4 | 1.2 | 0.4 | 4.0 |
| 100 | 164.8 | 8.1 | 0.7 | 0.4 | 179.5 | 16.3 | 4.1 | 0.3 |
| 200 | 205.2 | 1.9 | 0.8 | 1.2 | 247.0 | 19.6 | 1.2 | 0.3 |
| 300 | 227.9 | 1.4 | 2.2 | 0.5 | 233.0 | 7.8 | 2.7 | 0.8 |
| 500 | 203.5 | 2.5 | 2.9 | 0.7 | 240.7 | 2.1 | 2.4 | 0.0 |
| 750 | 179.1 | 1.0 | 2.0 | 0.6 | - | - | - | - |
| 1000 | 227.9 | 1.9 | 1.5 | 0.6 | - | - | - | - |
| 1500 | 280.3 | 15.2 | 1.0 | 0.9 | - | - | - | - |
| 2000 | 238.1 | 33.5 | 1.4 | 1.2 | 247.0 | - | 4.3 | - |

# Nitrate concentrations of river water

**Table S6** Photometric determination of nitrite, nitrate and ammonium on May 25^th^, 2022, in the river Rotbach on sampling points up to 2000 m downstream from the administration of ^15^N-enriched NH_4_Cl

| **Sampling point (m)** | **Nitrite (mg L^-1^)** | | **Nitrite (mg L^-1^)** | **Ammonium Nitrite (mg L^-1^)** |
| --- | --- | --- | --- | --- |
| 50 | | 0.053 | 7.173 | 0.142 |
| 100 | | 0.055 | 8.175 | 0.120 |
| 200 | | 0.056 | 7.545 | 0.142 |
| 750 | | 0.045 | 6.478 | 0.072 |
| 1500 | | 0.044 | 6.87 | 0.079 |
| 2000 | | 0.082 | 6.562 | 0.116 |

# References

1. Silva SR, Kendall C, Wilkison DH, Ziegler AC, Chang CCY. A new method for collection of nitrate from fresh water and the analysis of nitrogen and oxygen isotope ratios. *Journal of Hydrology.* 2000;228:22-36.DOI:10.1016/S0022-1694(99)00205-X.

2. Sigman DM, Casciotti KL, Andreani M, Barford C, Galanter M, Böhlke JK. A Bacterial Method for the Nitrogen Isotopic Analysis of Nitrate in Seawater and Freshwater. *Anal Chem.* 2001;73(4145-4153).DOI:10.1021/ac010088e.

3. McIlvin MR, Altabet MA. Chemical Conversion of Nitrate and Nitrite to Nitrous Oxide for Nitrogen and Oxygen Isotopic Analysis in Freshwater and Seawater. *Anal Chem.* 2005;77:5589-5595.DOI:10.1021/ac050528s.

4. Stange CF, Spott O, Apelt B, Russow RW. Automated and rapid online determination of 15N abundance and concentration of ammonium, nitrite, or nitrate in aqueous samples by the SPINMAS technique. *Isotopes Environ Health Stud.* 2007;43(3):227-236.DOI:10.1080/10256010701550658.

5. Russow R. Determination of 15N in 15N-Enriched Nitrite and Nitrate in Aqueous Samples by Reaction Continuous-flow Quadrupole Mass Spectrometry. *Rapid Commun Mass Spectrom.* 1999;13:1334-1338.DOI:10.1002/(SICI)1097-0231(19990715)13:13<1334::AID-RCM606>3.0.CO;2-C.

6. Eschenbach W, Lewicka-Szczebak D, Stange CF, Dyckmans J, Well R. Measuring (15)N Abundance and Concentration of Aqueous Nitrate, Nitrite, and Ammonium by Membrane Inlet Quadrupole Mass Spectrometry. *Anal Chem.* 2017;89(11):6076-6081.DOI:10.1021/acs.analchem.7b00724.

7. Eschenbach W, Well R, Dyckmans J. NO Reduction to N2O Improves Nitrate (15)N Abundance Analysis by Membrane Inlet Quadrupole Mass Spectrometry. *Anal Chem.* 2018;90(19):11216-11218.DOI:10.1021/acs.analchem.8b02956.

8. Dyckmans J, Eschenbach W, Langel R, Szwec L, Well R. Nitrogen isotope analysis of aqueous ammonium and nitrate by membrane inlet isotope ratio mass spectrometry (MIRMS) at natural abundance levels. *Rapid Commun Mass Spectrom.* 2021;35(10):e9077.DOI:10.1002/rcm.9077.

9. Huang K, Eschenbach W, Wei J, et al. Simultaneous 15N Online Analysis in NH4+, NO2–, NO3–, and N2O to Trace N2O Production Pathways in Nitrogen-Polluted Aqueous Environments. *ACS ES&T Water.* 2023;3(11):3485-3495.10.1021/acsestwater.3c00216.

10. Altabet MA, Wassenaar LI, Douence C, Roy R. A Ti(III) reduction method for one-step conversion of seawater and freshwater nitrate into N2O for stable isotopic analysis of 15N/14N, 18O/16O and 17O/16O. *Rapid Communications in Mass Spectrometry.* 2019;33(15):1227-1239.DOI:10.1002/rcm.8454.

11. Wassenaar LI, Douence C, Fortson S, Baer DS. Automated rapid triple-isotope (δ15N, δ18O, δ17O) analyses of nitrate by Ti(III) reduction and N2O laser spectrometry. *Isotopes in Environmental and Health Studies.* 2023;59(3):297-308.DOI:10.1080/10256016.2023.2222222.

12. Hilkert A, Böhlke JK, Mroczkowski SJ, et al. Exploring the Potential of Electrospray-Orbitrap for Stable Isotope Analysis Using Nitrate as a Model. *Analytical Chemistry.* 2021;93(26):9139-9148.DOI:10.1021/acs.analchem.1c00944.
